# Intravitreal bevacizumab versus intravitreal triamcinolone for diabetic macular edema–Systematic review, meta-analysis and meta-regression

**DOI:** 10.1371/journal.pone.0245010

**Published:** 2021-01-12

**Authors:** Mohamed Abdel-Maboud, Esraa Menshawy, Eshak I. Bahbah, Oumaima Outani, Amr Menshawy

**Affiliations:** 1 Faculty of Medicine, Al-Azhar University, Cairo, Egypt; 2 Faculty of Medicine, Al-Azhar University, New Damietta, Egypt; 3 Faculty of Medicine and Pharmacy of Rabat, Mohammed 5 University, Rabat, Morocco; Massachusetts Eye & Ear Infirmary, Harvard Medical School, UNITED STATES

## Abstract

**Background:**

The most frequent cause of vision loss from diabetic retinopathy is diabetic macular edema (DME). Earlier clinical trials tried to examine the role of intravitreal triamcinolone (IVT) and intravitreal bevacizumab (IVB) in DME; they either qualified IVT over IVB or IVB over IVT or did not exhibit a significant difference.

**Objective:**

This paper aims to compare the efficacy and safety of IVB versus IVT alone or combined IVB+IVT in the treatment of DME.

**Methods:**

We systematically searched PubMed, CENTRAL, Scopus, Embase, Science Direct, OVID, and Web of Science for randomized controlled trials of IVB versus IVT alone or combined IVB+IVT and IVT versus the combined IVB+IVT in DME patients.

**Results:**

A total of 1243 eyes of 17 trials were included in our meta-analysis and regression. Repeated injections of IVB were superior at improving VA comparing with those of IVT at 12, 24, 48-weeks, and IVB+IVT at 12, 24, 48-weeks. Single injections were comparable across the three arms regarding BCVA improvement. CMT reductions were also comparable across the three arms. Meanwhile, the overall safety regarding intraocular pressure and intraocular hypertension significantly favored the IVB group. Improvement in VA was best modified with CMT reduction from 480 um to 320um. This association was significant at 12-weeks in the three arms and persisted till 24-weeks and 48-weeks exclusively in the IVB group.

**Conclusions and relevance:**

Our analysis reveals that repeated successive injections associate with better BCVA compared to single injection. Current evidence affirms that IVB is superior to IVT and IVB+IVT at improving BCVA, comparable at reducing CMT, and presents a better safety profile in the treatment of DME.

## 1. Introduction

Diabetes mellitus (DM) affects over 422 million persons worldwide [1]. About 33% of patients with DM develop some form of diabetic-related eye damage [2]. For instance, the 10-year incidence of diabetic retinopathy in patients with T1DM is nearly 36%, while the 20-year incidence for T2DM is 84% in those taking insulin and 53% in those not taking insulin [3–5]. Diabetic retinopathy is a microvascular disorder correlated with the thickening of the peripapillary retinal nerve fiber layer [6]. The most frequent cause of vision loss from diabetic retinopathy is diabetic macular edema (DME), which can develop at any stage of retinopathy and is marked by edema and retinal thickening [7].

In DME chronic hyperglycemia upregulates vascular endothelial growth factor (VEGF), increasing vascular permeability, and angiogenesis [8, 9]. Additionally, a decent amount of evidence suggests that inflammatory mediators are partially engaged in the pathophysiology of DME and contribute substantially to the vascular permeability and the development of edema [10–12].

The potential treatments for DME involves macular laser photocoagulation (MLP), anti-VEGF agents, ocular corticosteroids, and pars plana vitrectomy [13]. MLP was the primary treatment for DME proving to be effective in limiting vision loss [14]. Over time the intravitreal injections of anti-VEGF agents have rapidly become the standard of care, due to its ability to adjust both visual and anatomical outcomes, besides the avoidance of laser-related complications like subretinal fibrosis and laser scars [15–17].

Being an ocular steroid, Triamcinolone performs an anti-inflammatory, anti-angiogenic, and cost-effective role in the treatment of DME; proved to be beneficial through several reports [18, 19]. Meanwhile, the humanized monoclonal antibody Bevacizumab presents promising anti-VEGF results working as an off-label therapeutic favorable option -for it is more affordable than most of the anti-VEGF agents. Comparatively, triamcinolone requires fewer injections, and a single intravitreal triamcinolone (IVT) injection might be as effective as three injections of intravitreal bevacizumab (IVB) for the treatment of DME [20]. This implies that IVT may reduce injection-related complications and improve patient compliance. However, the rates of intraocular pressure (IOP) increase and cataract development are expected to be higher in steroids-treated eyes [21, 22].

Earlier individual trials either qualified IVT over IVB or IVB over IVT or did not exhibit a significant difference [23–25]. Previous cumulative reviews tried to settle this controversy [26, 27], but with limited double-arm randomized controlled trials (RCTs) at the time, unreliable statistical methods, and short-term follow-ups; the debate is still unsettled.

In this systematic review, meta-analysis, and meta-regression of multiple-arm (RCTs): we compare the short and long-term safety and efficacy of IVB versus IVT alone or combined with IVB in the treatment DME, regarding visual acuity (VA), central macular thickness (CMT), IOP, intraocular hypertension (IOH), and pathogenesis factors (such as hypertension, diabetes duration, and HbA1C levels).

## 2. Materials and methods

We followed the Preferred Reporting Items for Systematic Reviews and Meta-analyses (PRISMA) statement guidelines [28], as well as the standards of the Cochrane Handbook for Systematic Reviews of Intervention [29].

### 2.1 Literature search strategy

The following electronic databases were comprehensively searched: PubMed, CENTRAL, Scopus, Embase, Science Direct, OVID, and Web of Science; using relevant keywords "diabetic macular edema", "DME", "bevacizumab", "Avastin", "triamcinolone", "IVB", "IVT" from inception till 20 August 2020. All published articles were considered with no restrictions in terms of language or publication period. Further, we manually scanned the bibliography of retrieved articles for additional relevant studies.

### 2.2 Eligibility criteria and study selection

We included studies with the following criteria: (a) randomized controlled trials (RCTs) that compared IVB of any dose with or without IVT of any dose; (b) patients of any age and sex, who had any type of diabetes, clinically significant DME and receiving naïve treatment; (c) reported data on any of the following outcomes: best-corrected visual acuity (BCVA) between 0.096 logMAR (Snellen = 20/25; ETDRS VALS = 80) and 1.3 logMAR (Snellen = 20/400; ETDRS VALS = 20), central macular thickness (CMT) > 300 μm defined by OCT machine, intraocular pressure (IOP), intraocular hypertension (IOH) at various weeks endpoints (4, 6, 12, 24, and 48-weeks); (d) Studies of additional injections or retreatment based on persistence of clinically significant macular edema according to Early Treatment Diabetic Retinopathy Study (ETDRS) criteria; (e) duplicated publications or reports were included once. Articles were excluded if: (I) non-randomized controlled trials or comparative interventional case series; (II) studies with DR without macular edema or studies of macular edema due to causes other than DR; (III) studies that compared IVB or IVT with different intervention, or studies that concerned with non-ocular outcomes or non-DME patients, as well as dissertations/thesis or animal studies; (IV) patients with macular edema related to recent intraocular surgery or other procedures, vitreous traction, history of any treatment for DR at any time or anticipating the need for pan retinal laser photocoagulation, existing/pre-existing glaucoma or ocular hypertension (IOP> 21 mmHg), steroid responders, recent history of arterial thromboembolic event, poorly controlled hypertension, or use of systemic steroids and/or systemic anti-VEGF agents. (II), (III) and (IV) were considered irrelevant during the screening process. Duplicates were removed using EndNote X7.1 software and retrieved references were screened in two step-wise manner: titles/abstracts screening for matching our inclusion criteria, followed by a full-text appraisal of relevant articles for eligibility to meta-analysis. Each step was performed by two independent reviewers.

### 2.3 Data extraction and risk of bias assessment

Each type of dataset was extracted independently by two authors. Discrepancies were settled through discussion and consensus among the reviewers. The extracted data involved the following: (1) study ID (name of the first author and year of publication), location, study design, major inclusion criteria, various intervention groups (arm, dosage, number of injections and the interval in-between), number of eyes, follow up duration and the conclusion of each study; (2) Baseline characteristics for each intervention arm of enrolled patients regarding age, sex, type of DM, insulin users (%), HbA1C level, hypertensive patients (%), and retinopathy severity (%); whether non-proliferative diabetic retinopathy (NPDR), proliferative diabetic retinopathy (PDR), or regressed PDR; (3) Risk of bias (ROB) domains; (4) Treatment outcome measures. The following outcome measures were extracted at 4, 6, 12, 24 and 48-weeks; to indicate the short, intermediate and long term efficacy related to the treatment groups: (I) means and standard deviations (SDs) of the different values for BCVA, CMT, and IOP at each endpoint following the intervention per eye; (II) number of eyes developed IOH of more than 21 mmHg IOP.

We adopted the Cochrane risk of bias (ROB) assessment tool, adequately described in chapter 8.5 of the Cochrane handbook [29]. ROB domains included Randomization (selection bias); Allocation concealment (selection bias); Blinding of participants (performance bias); Blinding of outcome assessment (detection bias); Incomplete outcome data (attrition bias), Selective reporting (reporting bias), and other sources of bias including unclear baseline characteristics and trial termination shortly due to data-dependent considerations. We classified RCTs in each domain as low, high, or unclear ROB as defined by Cochrane Handbook. Any discrepancies were resolved through discussion. The assessment of publication bias using the funnel plot and Egger’s test was also considered (Fig 3B). We also considered the Grading of Recommendations Assessment Development and Evaluation (GRADE) approach (Table 1).

**Table 1:** Shows the summary and baseline data of patients in included studies.

Author, Year and Setting	Study Design	Population	Treatment				No. of Eyes	Follow up	Female, (%)	Age (years)	Diabetes Mellitus		Insulin used (%)	HbA1C	HTN	IOP	BCVA (LogMAR)	CMT (μm)	Retinopathy severity			Conclusion
			Intervention	Dosage / 0.05ml	Number of injections	Interval = Weeks				(mean ± SD)	Duration (years)	Type II		(mean ± SD)	N (%)	(mean ± SD)	(mean ± SD)	(mean ± SD)	No. of Eyes (%)			
											(mean ± SD)								NPDR	Early PDR	Regressed PDR	
**Rodrigues et al. 2020**	RCT	Patients aged > 18 years with clinically significant DME, CST > 300 μm by SD-OCT, without any DME therapy and/or cataract surgery in the prior 4 months.	IVB	1.25 mg	10 to 13	4	33	48 weeks	19 (57)	60.7 ± 6.6	16.9 ± 8.0	NA	15 (46)	8.3	20 (60)	18.5 ± 0.3	0.50 ± 0.0	447.2 ± 24.4	33 (100)	0 (0)	0 (0)	• No difference between continued treatment with IVB versus IVT for DME treatment at week 48. • BCVA was stable in the IVB group, while IVT was associated with BCVA reduction from baseline and a higher risk of IOP elevation.
[Brazil]			IVT	1.20 mg/0.03 ml	7 to 9	4	32		20 (63)	62.8 ± 8.2	19.4 ± 9.1	NA	23 (71.5)	9.5	16 (50)	18.4 ± 0.2	0.60 ± 0.1	478.0 ± 19.7	32 (100)	0 (0)	0 (0)	
**Riazi-Esfahani et al. 2018**	RCT	Patients with bilateral clinically significant DME based on ETDRS criteria and CMT > 320 μm.	IVB	1.25 mg	72% -> 4	6	46	24 weeks	25 (54.3)	62 ± 8.6	NA	NA	NA	6.88 ± 1.28	0 (0)	NA	0.35±0.25	462± 124	46 (100)	0 (0)	0 (0)	• IVB/IVT show more significant reduction in CMT in early post-injection, but this effect was transient. • VA improvement was better in IVB group • Combination therapy may decrease the number of injections. • IVB/IVT was not accompanied with significant side effects.
[Iran]			IVB/IVT	1.25/2 mg	57% -> 4	6	46				NA	NA	NA		0 (0)	NA	0.38±0.3	466± 126	46 (100)	0 (0)	0 (0)	
**Neto et al. 2017**	RCT	Type 1 or 2 DM patients aged ≥ 18 years with clinically significant DME; BCVA of 20/40 to 20/400; a CMT ≥ 275 μm by SD-OCT; and no previous treatment with intravitreal injections, laser therapy, or surgery.	IVB	1.25 mg	3	4	39	24 weeks	16 (41)	NA	NA	32 (82.1)	25 (80.6)	NA	26 (74.3)	14.52	0.73	395.26	39 (100)	0 (0)	0 (0)	• Mono- or combination therapy was effective for DME treatment. • IVT alone or a drug combination may reduce the number of injections required when compared to IVB alone.
[Brazil]			IVT	4 mg	2	4	38		18 (47.4)	NA	NA	35 (92.1)	19 (65.5)	NA	26 (76.5)	14.34	0.77	407.33	38 (100)	0 (0)	0 (0)	
			IVB/IVT	1.25/2 mg	2	4	34		16 (47.1)	NA	NA	27 (84.4)	27 (93.1)	NA	23 (76.7)	14.65	0.83	412.6	34 (100)	0 (0)	0 (0)	
**Kasiri et al, 2017**	RCT	Patients with a diagnosis of diffuse DME who had never previously underwent treatment with laser therapy, intravitreal injections, or surgery. CMT ≥ 250 μm by SD-OCT.	IVB	IVB	1.25 mg	1	__	12 weeks	18 (60)	59.93 ± 5.32	NA	NA	NA	NA	NA	14.9 ± 2.9	0.63 ± 0.27	417 ± 141	30 (100)	0 (0)	0 (0)	• Both IVB and IVT may be effective in the treatment of diffused DME. • IVB may offer certain advantages over IVT in the short-term management of diffused DME.
[Iran]			IVT	IVT	4 mg	1	__		16 (53.33)	59.11 ± 9.51	NA	NA	NA	NA	0 (0)	14.4 ± 2.3	0.59 ± 0.31	451 ± 139	30 (100)	0 (0)	0 (0)	
**Sonoda et al, 2014**	RCT	Type 1 or 2 DM patients aged ≥ 18 years with a diagnosis of DME, CMT ≥ 250 by SD-OCT, BCVA of 0.097 to 1.0 logMAR, and HbA1C ≤ 12%.	IVB	1.25 mg	1	__	26	12 weeks	9 (36.62)	62.9 ± 11.4	12.7 ± 5.3	NA	NA	7.1 ± 1.1	17 (61.5)	13.1± 2.9	0.48 ± 0.32	495.7± 195.5	10 (38.46)	16 (61.54)	0 (0)	• The decrease in choroidal thickness in eyes with DME after IVT suggests that the choroidal pathology in diabetic retinopathy might be due to steroid-sensitive factors rather than vascular endothelial growth factor.
[Japan]			IVT	4 mg	1	__	25		8 (32)	59.2 ± 12.5	10.4 ± 4.8	NA	NA	7.0 ± 1.1	14 (56.0)	13.7± 2.4	0.39 ± 0.25	503.9± 171.4	10 (40)	15 (60)	0 (0)	
**Rakhee et al. 2014**	RCT	Type 2 DM patients with clinically significant DME and CMT > 300 μm.	IVB	1.25 mg	3	4	49	24 weeks	NA	54.73 ± 11.91	11.34 ± 6.69	49 (100)	NA	NA	0 (0)	15.10 ± 1.74	0.82 ± 0.14	478.10 ± 142.78	NA	NA	NA	• IVB/IVT is more effective than using IVB alone in the treatment of diffused DME. • Close IOP monitoring is required in patients treated with IVT.
[India]			IVB/IVT	1.25/2 mg	3	4	49		NA	58.18 ± 11.22	10.97 ± 4.65	49 (100)	NA	NA	0 (0)	15.26 ± 1.38	0.86 ± 0.09	474.71 ± 96.29	NA	NA	NA	
**Shoeibi et al. 2013**	RCT	Patients with refractory DME defined as macular edema not responsive to laser treatment.	IVB	1.25 mg	3	6	41	48–50 weeks	8/7*	60.4 ± 9.3	NA	NA	NA	9.7 ± 1.6	14 (34.1)	15.4 ± 2.8	0.88 ± 0.32	414.6 ± 62.1	30 (73.2)	3 (7.3)	8 (19.5)	• IVB have long-term beneficial effects for treatment of refractory DME. • Adding IVT to this regimen provides no additional long-term benefit.
[Iran]			IVB/IVT	1.25/2 mg	3	6	37		9/7*	59.1 ± 8.1	NA	NA	NA	9.6 ± 1.9	11 (29.7)	16.1 ± 2.2	0.92 ± 0.32	417.7 ± 139.4	31 (83.8)	0 (0)	6 (16.2)	
**Kriechbaum et al. 2013**	RCT	Patients with clinical significant DME because of systemic diabetes mellitus diagnosed for 43 months.	IVB	2.5 mg	3	4	15	48 weeks	18	59±11	NA	NA	NA	NA	NA	14.1 (13.2–15.1)**	0.3 (0.190–0.416)**	505 (437.9–571.7)**	15 (100)	0 (0)	0 (0)	• Both IVB and IVT are equally effective in reducing CMT in early DME. • After 6 months, rehabilitation of vision was comparable in both IVB and IVT. • After 12 months, BCVA was superior in the IVB than in the IVT.
[Austria]			IVT	8 mg	3	4	15									15.4 (14.4–16.5)**	0.32 (0.197–0.432)**	490 (433.2–546.7)**	15 (100)	0 (0)	0 (0)	
**Lim et al. 2012**	RCT	Patients with clinically significant DME based on ETDRS criteria, CMT of ≥ 300 μm by SD-OCT.	IVB	1.25 mg	1	__	38	48 weeks	19 (0.5)	61.4 ± 6.7	12.4 ± 4.5	NA	NA	7.4 ± 1.1	NA	15 ± 2	0.62 ± 0.23	447 ± 110	37 (97.4)	1 (2.6)	0 (0)	• IVB/IVT and IVT showed more pronounced effects during the earlier post injection period. • Levels of visual acuity or CMT at 12 months were comparable in the three study groups. • No beneficial effect of the combination injection was observed.
[South Korea]			IVT	4 mg	1	__	37		18 (48.65)	59.8 ± 7.9	13.0 ± 5.1	NA	NA	7.2 ± 1.2	NA	14 ± 1	0.65 ± 0.28	449 ± 106	37 (100)	0 (0)	0 (0)	
			IVB/IVT	1.25/2 mg	1	__	36		18 (0.5)	58.4 ± 5.9	12.5 ± 5.4	NA	NA	7.5 ± 1.2	NA	14 ± 3	0.64 ± 0.5	458 ± 92	35 (97.2)	1 (2.8)	0 (0)	
**Soheilian et al. 2012**	RCT	Patients with clinically significant DME based on ETDRS criteria.	IVB	1.25 mg	2	12	50	96 weeks	27 (54)	60.5 ± 5.9	10.5 ± 3.2	NA	NA	NA	0 (0)	16.7 ± 2.4	0.71 ± 0.28	341 ± 148	46 (98)	4 (2)	0 (0)	• VA improvement was better in IVB group than combined IVB/IVT at Month 6 that did not sustain up to 24 months. • IVB treatment may be a better choice than combined IVB/IVT in short term, the magnitude of this beneficial effect diminishes over time.
[Iran]			IVB/IVT	1.25/2 mg	2	12	50		22 (44)	62.3 ± 6.8	10.4 ± 2.6	NA	NA	NA	0 (0)	14.4 ± 2.6	0.73 ± 0.28	359 ± 137	48 (96)	2 (4)	0 (0)	
**Marey et al. 2011**	RCT	Patients with clinically significant macular edema based on ETDRS criteria.	IVB	1.25 mg	1	__	30	12 weeks	14 (46.67)	57.60 ± 7.30	NA	NA	NA	NA	0 (0)	15.47 ± 2.93	0.22 ± 0.12	445.06 ± 123.87	44 (48.89).	46 (51.11)	0 (0)	• IVB is an effective for treatment of DME, and has a long lasting effect when compared with IVT or combined IVT/IVB. • Adding IVT does not affect the outcome measures except for elevating the IOP in treated patients in the early post-injection period.
[Egypt]			IVT	4 mg	1	__	30		12 (40)	57.66 ± 7.19	NA	NA	NA	NA	0 (0)	14.83 ± 2.34	0.18 ± 0.12	492.30 ± 145.91				
			IVB/IVT	1.25/2 mg	1	__	30		11 (36.67)	57.66 ± 7.44	NA	NA	NA	NA	0 (0)	15.67 ± 2.86	0.19 ± 0.13	477.70 ± 153.38				
**Shahin et al. 2010**	RCT	Patients with diffuse macular edema not associated with vitreomacular traction.	IVB	1.25 mg	1	__	24	12 weeks	20 (83.3)	52.7	NA	NA	NA	NA	NA	NA	NA	NA	NA	NA	NA	• IVT appears to be more effective treatment for diabetic macular edema than IVB. • Short term outcomes indicate that IVB was not associated with surgical complications compared to IVT.
[Egypt]			IVT	4 mg	1	__	24				NA	NA	NA	NA	NA	NA	NA	NA	NA	NA	NA	
**Isaac et al. 2009**	RCT	Type 2 DM patients with DME with or without previous photocoagulation with CMT ≥ 300 μm by SD-OCT, and blood pressure < 160/90 mmHg.	IVB 1.25 mg	1.25 mg	1	__	11	24 weeks	5 (45.45)	64.6 ± 9.75	NA	11 (100)	NA	NA	NA	NA	0.72 ± 0.3	528 ± 105	11 (100)	0 (0)	0 (0)	• IVT appears to be more efficient in reducing DME, providing longer lasting visual improvement, relative to IVB. • Eyes treated with IVT had the highest percentage increase in IOP.
[Brazil]			IVT 4 mg	4 mg	1	__	11				NA	11 (100)	NA	NA	NA	NA	0.72 ± 0.23	453 ± 88	11 (100)	0 (0)	0 (0)	
**Soheilian et al. 2009**	RCT	Patients with clinically significant DME based on ETDRS criteria.	IVB	1.25 mg	22% -> 2	6	50	36 weeks	27 (54)	60.5 ± 5.9	10.5 ± 3.2	NA	NA	NA	0 (0)	16.7 ± 2.4	0.71 ± 0.28	341 ± 149	46 (98)	4 (2)	0 (0)	• IVB injection in patients with DME shows a better visual outcome at 24 weeks. • A change in CMT beyond the 6-week time point that corresponded to the vision change was not detected. • No adjunctive effect of IVT was demonstrated.
[Iran]			IVB/IVT	1.25/2 mg	22% -> 2	6	50		22 (44)	62.3 ± 6.8	10.4 ± 2.6	NA	NA	NA	0 (0)	14.4 ± 2.6	0.73 ± 0.28	359 ± 137	48 (96)	2 (4)	0 (0)	
**Ahmadieh et al. 2008**	RCT	Eyes with clinically significant DME unresponsive to previous macular laser photocoagulation, with the last session being more than 3 months prior.	IVB	1.25 mg	3	6	41	24 weeks	NA	59.7 ± 8.3	NA	NA	**37**	9.95	31 (30.7)	NA	NA	NA	94 (81.7)	5 (4.3)	16 (13.9)	• IVB had a beneficial effect on refractory DME in terms of CMT reduction and BCVA improvement. • Addition of IVT in the first injection seemed to induce earlier visual improvement; however, it did not show any significant additive effect later during follow-up.
[Iran]			IVB/IVT	1.25/2 mg	3	6	37						**38**	9.35								
**Faghihi et al. 2008**	RCT	Type 2 DM patients with clinically significant DME, BCVA ≤ 20/40 (ETDRS chart) (≤ 0.3 logMAR) and CMT ≥ 250 μm.	IVB	1.25 mg	1	__	42	16 weeks	19 (45.2)	59 ± 6	NA	42 (100)	NA	NA	0 (0)	15 ± 2	0.70 ± 0.31	356 ± 116	NA	NA	NA	• IVB or combined IVB/IVT injection show significantly macular thickness reduction in diabetic patients. • The response for IVB alone was short-lived. • Reduction in macular thickness was only marginally associated with visual acuity improvement in the IVB/IVT injection group.
[Iran]			IVB/IVT	1.25/2 mg	1	__	41		22 (53.7)	56 ± 7	NA	41 (100)	NA	NA	0 (0)	14 ± 1	0.77 ± 0.33	387 ± 154	NA	NA	NA	
**Paccola et al. 2007**	RCT	Patients with clinically significant refractory DME, BCVA ≤ 20/40 (ETDRS chart) (≤ 0.3 logMAR) and CMT ≥ 300 μm.	IVB	1.25 mg	1	__	13	24 weeks	6 (46.0)	65.58 ± 8.44	12.33 ± 5.34	NA	6 (46)	8.65 ± 0.85	0 (0)	14 ± 0.49***	0.9375 ± 0.0615***	466 ± 38.16***	2 (15)	NA	NA	• IVT may offer certain advantages over IVB in the short-term management of refractory DME, specifically with regard to changes in CMT.
[Brazil]			IVT	4 mg	1	__	13		5 (37.5)	67.08 ± 4.67	12.66 ± 5.69	NA	6 (46)	8.79 ± 0.88	0 (0)	14.91 ± 0.77***	0.9366 ± 0.0569***	440.33 ± 36.14***	3 (23)	NA	NA	

Abbreviations: RCT = randomized controlled clinical trials; DM = diabetes mellitus; DME = diabetic macular edema; CMT = central macular thicknes; BCVA = best-corrected visual acuity; logMAR = logarithm of the minimum angle of resolution; NPDR = non-proliferative diabetic retinopathy; PDR = proliferative diabetic retinopathy

*: F/M

**: means with CI

***: means with SEM.

### 2.4 Data analysis

Statistical analysis was performed using Open Meta[Analyst] package from Brown University—School of Public Health. Applying the random-effects model with Der-Simonian Liard method: continuous data of means and standard deviations were pooled as weighted mean differences (MD), dichotomous data of event-total were calculated as relative risks (RR). Subsequently, the MD (VA, CMT, IOP) and RR (IOH) among the three arms (IVB vs. IVT or IVB vs. IVT+IVB or IVT vs. IVT+IVB) were analyzed and provided a 95% confidence interval (CI). Missing SD of mean change from baseline was calculated from the standard error or 95% CI. To test for statistical heterogeneity between trials Chi-square and I2 tests were performed; values of 0%-40%, 30%-60%, 50%-90%, and 75%-100% represented low, moderate, substantial, and considerable heterogeneity, respectively according to Cochrane Handbook of Systemic Review and Meta- analysis. P<0.1 was set as a level of significant heterogeneity. When significant heterogeneity was detected, we conducted a sensitivity analysis to find the source of heterogeneity by excluding one study at a time. Subgroup analysis according to study arms and repeated injections was also performed. Additionally, a meta-regression was employed to examine whether injections, sex, age, hemoglobin A1C level, diabetes duration, insulin usages, hypertension, or degree of retinopathy may predict alterations in VA and CMT.

## 3 Results

### 3.1 Search results and characteristics of included studies

**Fig 1:**
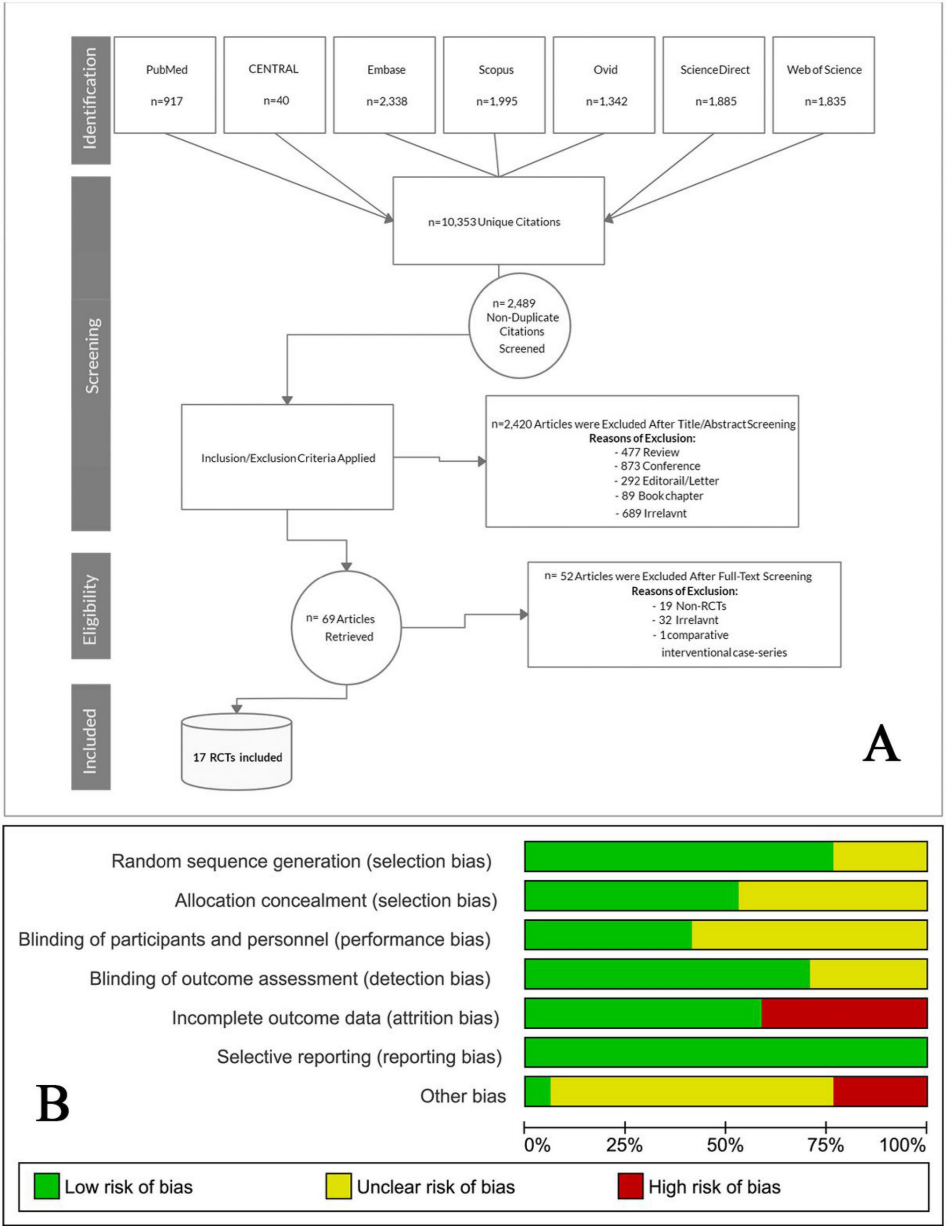
**A**. PRISMA flow diagram illustrates the search strategy, screening and the selection process. **B**. Risk of bias graph according to Cochrane risk of bias assessment tool.

Our search retrieved 10353 unique citations from searching electronic databases. Following title and abstract screening, 69 full-text articles were retrieved and screened for eligibility. Of them, 52 articles were excluded, and 17 RCTs (n = 1243 eyes) were reviewed in detail and included in this meta-analysis (PRISMA flow diagram; Fig 1A) [23, 24, 30–44]. The references of the included RCTs were manually searched, but no further reports were added. All of the included studies were performed between 2007–2020, seven studies in Iran [31–33, 36, 42–44], four studies in Brazil [23, 34, 38, 39], two studies in Egypt [35, 40], and one study in Japan [30], India [37], Australia [41], and South Korea [24]. Seven studies compared IVB and IVT alone [23, 30, 34, 35, 38, 41, 42], 10 studies compared IVB vs. IVB+IVT [24, 31–33, 36, 37, 39, 40, 43, 44] and three studies compared IVT vs. IVB+IVT [24, 39, 40]. The majority of the studies injected 1.25 mg of IVB, 4 mg of IVT, and 1.25/2 mg of IVB/IVT. The follow-up period ranged from 12 to 96 weeks. Both sexes were represented equally in each study. Table 2 summarize the characteristics of included patients and studies. It is worth mentioning that Neto et al. did not report the SD values of the outcomes, so the study could not be included in the pooled analysis [39].

**Table 2:** Shows the GRADE framework for the major outcomes.

Certainty assessment							№ of eyes		Effect		Certainty	Importance
№ of studies	Study design	Risk of bias	Inconsistency	Indirectness	Imprecision	Other considerations	IVB	IVT or IVB+IVT	Relative (95% CI)	Absolute (95% CI)		
**LogMAR BCVA (follow up: 48 weeks; Scale from: −0.044 to 0.010)**												
14	randomised trials	not serious	serious ^a^	not serious	not serious	dose response gradient	444	562	-	MD **0.089 1000 lower** (0.107 lower to 0.07 lower)	⨁⨁⨁⨁ HIGH	CRITICAL
**CMT (follow up: 48 weeks; Scale from: −7.267, to 19.085)**												
14	randomised trials	not serious	serious ^a^	not serious	serious ^b^	all plausible residual confounding would suggest spurious effect, while no effect was observed	444	562	-	MD **5.909 1000 more** (7.267 fewer to 19.085 more)	⨁⨁⨁◯ MODERATE	IMPORTANT
**IOP (follow up: 48 weeks; Scale from: −1.151 to −0.174)**												
5	randomised trials	not serious	not serious ^c^	not serious	serious ^c^	none	169	140	-	MD **0.662 1000 lower** (1.151 lower to 0.174 lower)	⨁⨁⨁◯ MODERATE	CRITICAL
**IOH (follow up: 48 weeks)**												
7	randomised trials	not serious	serious ^c^	not serious	serious ^c^	strong association	0/216 (0.0%)	22/276 (8.0%)	**RR 0.319** (0.120 to 0.842)	**54 fewer per 1,000** (from 70 fewer to 13 fewer)	⨁⨁⨁◯ MODERATE	CRITICAL

**Question**: Should IVB vs. IVT or IVB+IVT be used for DME?

**CI:** Confidence interval; **MD:** Mean difference; **RR:** Risk ratio

Explanations

a. Six included studies reported superiority of IVT or combined IVB+IVT compared with IVB alone. Eight other trials demonstrated that IVB was more efficient in reducing DME relative to IVT or IVB+IVT. Another three trials reported that the two drugs didn't differ markedly in terms of their effects in improving VA and reducing CMT.

b. The reduction was not significant regarding CMT during the early, intermediate, and late follow-ups (up to 48 weeks). Even though a slight superiority was present for IVB at 4-weeks, IVB+IVT at 12-weeks, and IVT at 24-weeks; the very wide 95% CI of these findings exclude it from clinical significance.

c. Wide 95% CI was present at some endpoints.

### 3.2 The potential source of bias

According to the Cochrane ROB tool, the quality of the included studies was from moderate to high. The main concern was incomplete outcome data (loss of follow-up), which was determined in Rodrigues et al. [34], Riazi-Esfahani et al. [36], Sonoda et al. [30], Lim et al. [24], Soheilian et al. [31, 32], and Paccola et al. [38]. A summary of quality assessment domains is shown in Fig 1B while authors’ judgments with justifications are shown in S1 File, S1 Fig.

### 3.3 Outcomes

#### 3.3.1 CMT

**Fig 2:**
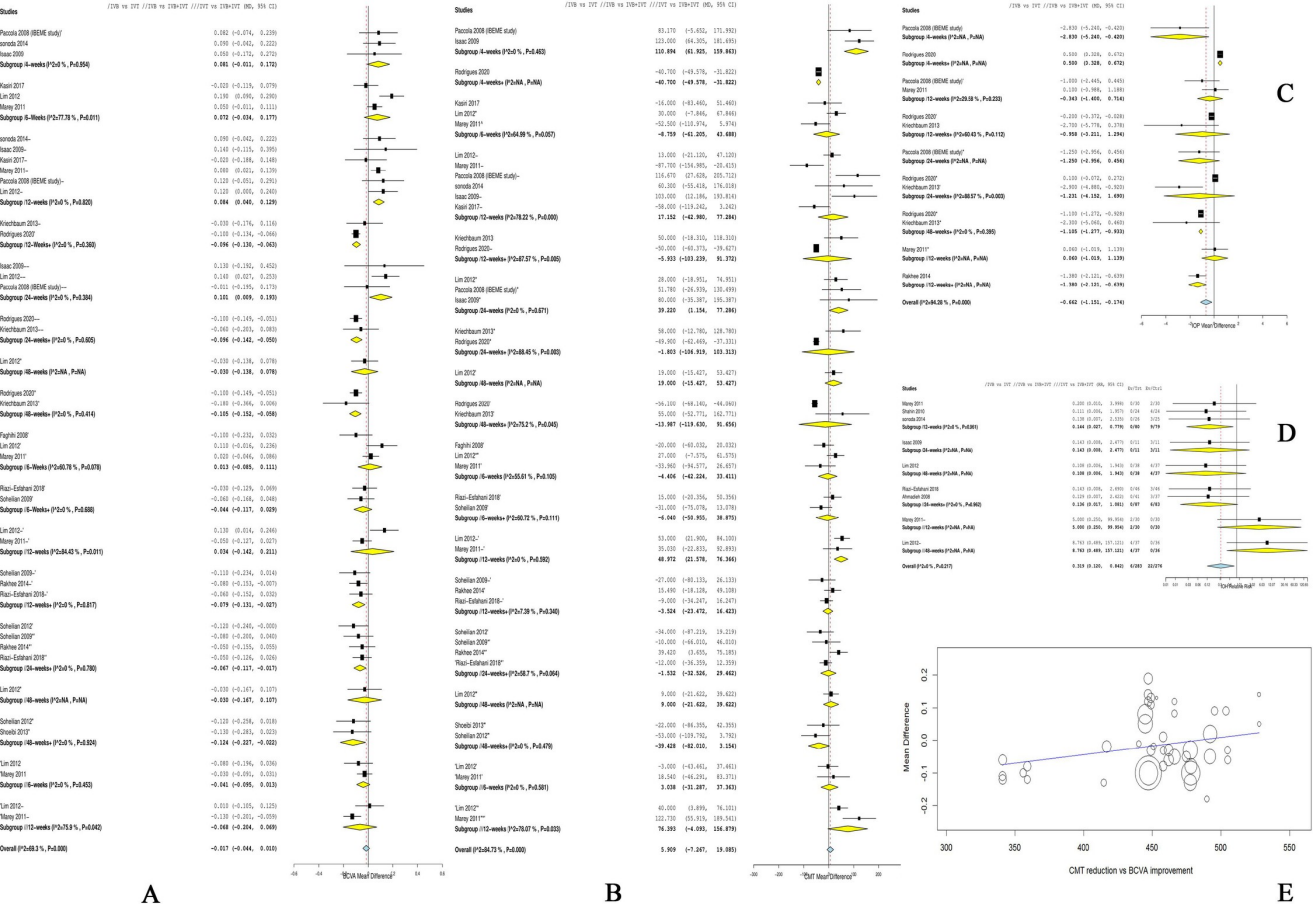
**A**. Forest plot shows the mean difference (MD) in BCVA (logMAR) along with the associated 95% CI in the three arms: (/) indicates IVB vs. IVT, (//) indicates IVB vs. IVB+IVT, (///) indicates IVT vs. IVB+IVT, and (+) indicates repeated injections; at 4, 6, 12, 24, and 48 weeks. **B**. Forest plot shows the mean difference (MD) in CMT (um) along with the associated 95% CI in the three arms: (/) indicates IVB vs. IVT, (//) indicates IVB vs. IVB+IVT, (///) indicates IVT vs. IVB+IVT, and (+) indicates repeated injections; at 4, 6, 12, 24, and 48 weeks. **C**. Forest plot shows the mean difference (MD) in IOP (mmHg) along with the associated 95% CI: (/) indicates IVB vs. IVT, (//) indicates IVB vs. IVB+IVT, and (+) indicates repeated injections; at 4, 12, 24, and 48 weeks. **D**. Forest plot shows the risk ratio (RR) of IOH along with the associated 95% CI in the three arms: (/) indicates IVB vs. IVT, (//) indicates IVB vs. IVB+IVT, (///) indicates IVT vs. IVB+IVT, and (+) indicates repeated injections; at 12, 24, and 48 weeks. **E**. Double interaction regression between CMT mean difference on x-axis and BCVA mean difference on y-axis.

*3*.*3*.*1*.*1 IVB vs*. *IVT*. The overall effect showed no significant difference between the two groups in CMT after 4 weeks (MD = 51.76, 95% CI [-71.55; 175.07]), 6 weeks (MD = -8.75, 95% CI [-61.20; 43.68]), 8 weeks (MD = 14.49, 95% CI [-106.85; 135.84]), 12 weeks (MD = 8.47, 95% CI [-36.53; 53.48]), 24 weeks (MD = 24.96, 95% CI [-36.05; 85.99]), 36 weeks (MD = -12.09, 95% CI [-102.48; 78.29]), and 48 weeks (MD = -5.00, 95% CI [-72.28; 62.26]). Pooled analyses were heterogeneous; therefore, sensitivity analysis was applied when applicable, yet presented no difference. (Heterogeneity values are reported in Fig 2B).

*3*.*3*.*1*.*2 IVB vs*. *IVB/IVT*. Pooled analysis of five studies [24, 32, 36, 40, 43] showed no significant difference between the two groups in CMT after 6 weeks (MD = 51.76, 95% CI [-71.55; 175.07]). Pooled analysis was homogenous (I^2^ = 43.6%, p = 0.13).

After 12 weeks, we could not find any significant difference between both groups (MD = 14.16, 95% CI [-14.70; 43.03]). Pooled analysis was heterogeneous (I^2^ = 66.7%, p = 0.02). Heterogeneity was best resolved by subgrouping into single and repeated injections, yet presented no difference, Fig 2B.

The overall effect of four studies [31, 32, 36, 37] showed no significant difference between the two groups in CMT after 24 weeks (MD = -1.53, 95% CI [-32.52; 29.46]). Pooled data were heterogonous (I^2^ = 58.7%, p = 0.06). Heterogeneity was best resolved by excluding the study of Rakhee et al. [37], (I^2^ = 0%, p = 0.75). This statistical heterogeneity could be due to the fact that most studies repeated the injections each 6 weeks, except for Rakhee et al. where they repeated the injection each 4 weeks. Following resolving heterogeneity, the effect estimate showed an insignificant reduction in the CMT in the IVB group (MD = -15.02, 95% CI [-35.62; 5.57]).

In terms of the 48-week period, a pooled analysis of three studies [24, 31, 33] showed that there was no significant difference between both groups (MD = -15.64, 95% CI [-54.38; 23.10]), with homogenous data (I^2^ = 47%, p = 0.15).

*3*.*3*.*1*.*3 IVT vs*. *IVB/IVT*. The overall effect of two studies [24, 40] showed no significant difference between the two groups in CMT after 6 weeks and 12 weeks (MD = 3.03, 95% CI [-31.28; 37.36], and MD = 76.39, 95% CI [-4.09; 156.87], respectively) (Heterogeneity values are reported in Fig 2B).

#### 3.3.2 LogMAR BCVA

**Fig 3:**
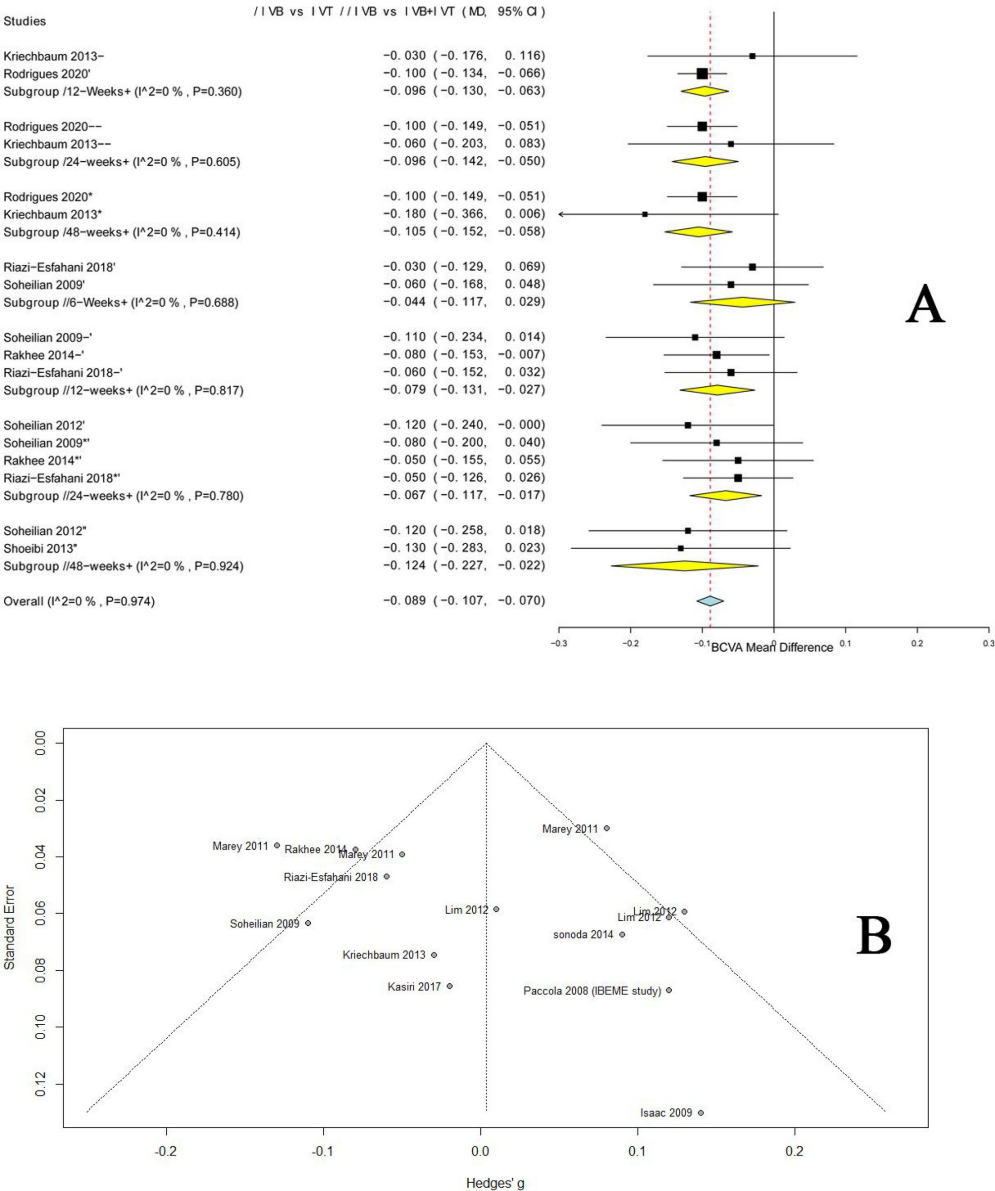
**A**. Forest plot shows the mean difference (MD) in BCVA (logMAR) along with the associated 95% CI in of the repeated injections’ groups among the three arms: (/) indicates IVB vs. IVT, (//) indicates IVB vs. IVB+IVT, (///) indicates IVT vs. IVB+IVT, and (+) indicates repeated injections; at 6, 12, 24, and 48 weeks. **B**. Funnel plot of BCVA showing no evidence of publication bias.

*3*.*3*.*2*.*1 IVB vs*. *IVT*. In terms of the overall effect, no significant difference was noted between the two groups in LogMAR BCVA after 4 weeks (MD = 0.08, 95% CI [-0.01; 0.17]), 6 weeks (MD = 0.07, 95% CI [-0.03; 0.18]), and 24 weeks (MD = 0.002, 95% CI [-0.12;0.12]). Pooled analyses were homogenous for 4-week period (I^2^ = 0%, p = 0.95), and heterogeneous for 6-week period and 24-week period (I^2^ = 77.8%, p = 0.01 and I^2^ = 75.3%, p<0.01, respectively). Heterogeneity was best resolved by excluding the study of Lim et al. [24], (I^2^ = 28%, p = 0.24 and I^2^ = 0%, p = 0.42). A main explanation for this statistical heterogeneity is that Lim et al. considered repeated injections of IVB only with no repetition of IVT injections even in the combination arm. After performing the sensitivity analysis, IVB significantly decreased the LogMAR BCVA more than IVT after 24 weeks (MD = -0.09, 95% CI [-0.13; -0.04]). Regarding the 48-week period, IVB demonstrated a significant reduction in the LogMAR BCVA (MD = -0.09, 95% CI [-0.17; -0.01]). Pooled data were homogenous (I^2^ = 0%, p = 0.55). Comparatively, in the subgroup of repeated injections: IVB significantly decreased the LogMAR BCVA after 12 weeks (MD = −0.096, 95% CI [−0.130; −0.063]), 24 weeks (MD = −0.096, 95% CI [−0.142; −0.050]), 48 weeks (MD = −0.105, 95% CI [−0.152; −0.058]), and the pooled data were homogenous (Heterogeneity values are reported in Figs 2A and 3A).

*3*.*3*.*2*.*2 IVB vs*. *IVB/IVT*. The pooled analysis revealed no significant difference between the two arms in LogMAR BCVA after 6 weeks (MD = -0.01, 95% CI [-0.07; 0.05]) and 12 weeks (MD = -0.04, 95% CI [-0.11; 0.03]). The analyses were homogenous for 6-week period (I^2^ = 42.6%, p = 0.14), and heterogeneous for 12-week period (I^2^ = 62.8%, p = 0.03). Heterogeneity was best resolved by excluding the study of Lim et al. [24], (I^2^ = 0%, p = 0.85). The explanation of this statistical heterogeneity of Lim et al. has been provided in section 3.3.2.1. After performing the sensitivity analysis, IVB significantly decreased the LogMAR BCVA after 12 weeks (MD = -0.07, 95% CI [-0.11; -0.03]). Similarly, IVB showed a significant reduction in the LogMAR BCVA after 24 weeks and 48 weeks (MD = -0.07, 95% CI [-0.1172; -0.0174]; MD = -0.09, 95% CI [-0.17; -0.01] respectively, and the pooled data were homogenous. Further, in the subgroup of repeated injections: IVB significantly decreased the LogMAR BCVA after 12 weeks (MD = −0.079, 95% CI [−0.131; −0.027]), 24 weeks (MD = −0.067, 95% CI [−0.117; −0.017]), 48 weeks (MD = −0.124, 95% CI [−0.227; −0.022]), and the pooled data were homogenous. (Heterogeneity values are reported in Figs 2A and 3A).

*3*.*3*.*2*.*3 IVT vs*. *IVB/IVT*. The overall effect of two studies [24, 40] showed no significant difference between the two groups in the LogMAR BCVA after 6 weeks and 12 weeks (MD = -0.04, 95% CI [-0.09; 0.01], and MD = -0.07, 95% CI [-0.20; 0.07], respectively). (Heterogeneity values are reported in Fig 2A).

### 3.4 Safety outcome

#### 3.4.1 IOP

*3*.*4*.*1*.*1 IVB vs*. *IVT*. In terms of IOP, both groups were comparable at 12 weeks with single and repeated injections (MD = −0.343, 95% CI [−1.400; 0.714]; and MD = −0.958, 95% CI [−3.211, 1.294], respectively), and 24 weeks with single and repeated injections (MD = −1.250, 95% CI [−2.956, 0.456]; and MD = −1.231, 95% CI [−4.152, 1.690], respectively). Also, no definitive conclusion can be drawn at the 4-weeks follow-up either with single or repeated injections, for only one study was available in each analysis. However, IOP was significantly lower in the IVB group after 36 weeks (MD = -2.3972 [-2.7040; -2.0904]) and 48 weeks (MD = -1.1047 [-1.2766; -0.9327]). Pooled data were homogenous. (Heterogeneity values are reported in Fig 2C).

*3*.*4*.*1*.*2 IVB vs*. *IVB/IVT*. The pooled analysis of two [24, 40] studies showed that both groups were comparable in terms of IOP after 12 weeks (MD = -0.72, 95% CI [-2.12; 0.69]). Pooled analysis was heterogeneous, Fig 2C.

*3*.*4*.*1*.*3 IVT vs*. *IVB/IVT*. No data comparing IVT vs. IVB+IVT were available to analyze.

#### 3.4.2 IOH

*3*.*4*.*2*.*1 IVB vs*. *IVT*. The overall effect of five studies [23, 24, 30, 35, 40] showed that IVB significantly associated with a lower risk of IOH compared to IVT (RR = 0.03, 95% CI [0.02; 0.04]). Pooled data were homogenous (I^2^ = 0, p = 1.00), Fig 2D.

*3*.*4*.*2*.*2 IVB vs*. *IVB/IVT*. The overall effect of two studies [36, 44] showed that IVB significantly associated with a lower risk of IOH compared to IVB/IVT (RR = 0.03, 95% CI [0.02; 0.06]). Pooled data were homogenous (I^2^ = 0, p = 0.98), Fig 2D.

*3*.*4*.*2*.*3 IVT vs*. *IVB/IVT*. The overall effect of two [24, 40] studies showed that the risk of IOH was higher in the IVT group compared to the IVB/IVT group (RR = 29.04, 95% CI [0.49; 1712.20]); however, the effect estimate was not significant. Pooled data were homogenous (I^2^ = 0, p = 0.89), Fig 2D.

### 3.5 Meta-regression models

Results from multiple regression models showed that the rates of BCVA, CMT and IOP were significantly modified by sex, DM duration, insulin use, HbA1C levels, hypertension (HTN); this combinations yielded R^2^ 100% (Coefficients 0.0226399, -0.2665421, 0.0804644; P = 0.0083) S2A–S2C Fig. The range of 1.5–4.8 IVB injections predicts more promising improvement in VA and reduction in CMT with 70% R^2^ (Coefficients -.0150974, -7.519123; P = 0.0003) S2D Fig. Double interaction regression between CMT and BCVA revealed favorable association with CMT reduction from 480 um to 320um (Coefficients .0005339; P = 0.0835), Fig 2E. This association was significant at 12-weeks in the three arms and persisted till 24-weeks and 48-weeks exclusively in the IVB group (Coefficients -0.144, -0.124, -0.165; P = 0.009).

## 4 Discussion

In this systematic review and meta-analysis of 17 RCTs and 1243 eyes: six of our included studies reported superiority of IVT or combined IVB+IVT compared with IVB alone in the treatment of DME [23, 24, 31, 38, 43]. However, eight other trials demonstrated that IVB was more efficient in reducing DME relative to IVT or IVB+IVT [32, 33, 35, 37, 39–41, 44]. To complicate this even further: three other trials reported that the two drugs did not differ markedly in terms of their effects in improving VA and reducing CMT [34, 36, 42]. In a previous meta-analysis of 6 RCTs by Zhang et al.: IVT was superior in improving short-term VA and reducing long-term CMT [26]. Nonetheless, the relatively small sample size, short term follow-ups, absence of repeated-dose consideration, fixed-effect model reliance and substantial heterogeneity left the question unanswered. Which treatment is more efficient remains a valid debate. Thus, we performed this meta-analysis to compare the efficacy and safety of IVB with IVT alone or combined IVB+IVT in DME patients. We considered the long-term follow-ups, the effect of multiple injections, and the possible associations between the underlying pathogenesis and the drug's mechanisms of action. It's important to note that we could not include the work of Shimura et al. in our final analysis [45]. Though it was included as an RCT in the previous meta-analysis, we found no characteristics of an RCT design in the original manuscript. Attempts to contact the authors for clarification received no response, so we excluded the study.

In our analysis: we found that the group who received repeated injections of IVB had a statistically significant improvement in BCVA over the relative IVT and IVT+IVB groups at 12, 24, and 48-weeks follow-up. Still, the three groups were comparable regarding CMT reduction as the difference was not significant during the early, intermediate, and late follow-ups (up to 48 weeks). Even though a slight superiority was present for IVB at 4-weeks, IVB+IVT at 12-weeks, and IVT at 24-weeks; the very wide CI of these findings exclude it from clinical significance. Although IVT presented a slight increase at 12 and 24-weeks, the wide confidence interval (CI) yield it clinically insignificant. CMT reductions were also comparable across the three arms. These findings reveal that there is no independent correlation between anatomical change (CMT) and functional change (BCVA). Our meta-regression for injections showed no favorable overlap for both BCVA and CMT, which could indicate that no single regimen can guarantee both increase in VA and decrease in CMT at the same time. But this should not be the case at certain specific ranges and injections. The double interaction regression between the two outcomes VA & CMT with subgroup consideration revealed that improvement in VA was best associated with CMT reduction from 480um to 320um. This association was significant at 12-weeks in the three arms and persisted till 24-weeks and 48-weeks exclusively in the IVB group. This further solidifies the multifactorial idea that age, hemoglobin A1C level, diabetes duration, insulin usages, and degree of retinopathy proliferation are all responsible for the change in VA and CMT [25, 46]. Moreover, the different degrees of macular ischemia could explain why some patients have no significant improvement in vision despite the reduction of thickness.

Attempting to analyze this multivariate pathogenesis, we considered performing additional meta-regression analysis. The duration of DM, insulin usages, levels of HbA1C, and HTN were all inversely associated with visual outcomes. According to this regression, the type of patient who responds best on treatment is a diabetic female with mild or no HTN with a short history of DM-II <10 years, HbA1C <8%, and low or no insulin intake. The interaction regression of this combination yields an R^2^ of 100%. Other factors like age, and degree of retinopathy proliferation do not seem to affect the outcomes as much.

Our findings exhibit a favorable response to IVB compared with IVT or IVB+IVT in improving VA up to 48-weeks. The reason why this difference did not persist with the single injection can be attributed to the limited effective duration of these injections. At first glance, the statistical insignificance between the two drugs regarding CMT reduction may indicate an equivalent share within VEGF angiogenesis and inflammatory transduction proposed mechanisms. However, the correlation between the two mechanisms appears to be non-linear, as the combined IVB+IVT also presented no statistical difference. This finding could either indicate that antagonizing multiple mechanisms simultaneously may lead to more resistance and less improvements, or that another unclear balancing factor could be compromised by this combination. Either way, this critical relation needs further investigation in the future pathological and pharmacological studies. Still, the 12-weeks improvement of VA surpasses the traditional pharmacological data that estimated a single IVB injection as effective only for 6 weeks [47, 48]. Also, it defies the prevailing assumption that an IVT injection better improves VA in the first 12 weeks of follow-up; and the presumption that a single IVT injection can be comparable to three IVB injections [20, 49, 50]. Some reports extend this even further, in a 96 weeks follow-up: Soheilian et al. 2012 reported a significant superiority of the IVB over the combined IVB+IVT up to 24 weeks [31].

The majority of our included studies used a standard dose of 1.25mg/0.05ml for IVB, 4mg/0.05ml for IVT, 1.25+2mg/0.05ml for IVB+IVT. Nine studies considered repeated injections and retreatments at different intervals, ranging from 4–12 weeks. Meta-regression revealed that repeated successive injections associate with better VA, and the range of 1.5–4.8 injection predicts more promising improvement in VA and reduction in CMT. This further supports the idea of dose-response proportional efficacy; but still promotes the idea of fewer injections as possible, to guarantee a lower incidence of injection-related complications such as endophthalmitis, high IOP, and weak patient compliance [20, 51]. A possible explanation for why the efficacy is not better in over 4.8 injections could be due to the fact that some eyes show low-response to treatment with regard to VA gain and CMT reduction, as pointed out by Menke et al.; 30% of patients showed low response to ranibizumab after 4 weeks [52].

A higher level of intraocular VEGF is considered a major pathogenic factor in DME, and a contributor to the increased IOP [53, 54]. Thus, measuring the long-term change in the IOP is critical in the assessment of drugs' safety and efficacy. In our findings: the group who received IVB had a statistically significant lower IOP than the group who received IVT at 36 and 48 weeks follow-up. Additionally, the incidence of IOH was significantly lower in the IVB group in comparison with IVT or IVB+IVT. This effect seems to persist even with different IVB and IVT injections. For instance, Rodrigues et al. considered 10–13 repeated injection of 1.25mg/0.05ml IVB and 7–9 repeated injection of 1.20mg/0.03ml IVT; Lim et al. considered repeated injection of IVB only with no repetition of IVT -even in the combination arm, and Kriechbaum et al. considered 3 injections of 2.5mg IVB and 8mg IVT [24, 34, 45]. Their results were consistent with our findings, together with the results of the previous meta-analysis of Zhang et al.

Meanwhile, the effect of combined laser with IVB or IVT is still unclear. Pappas et al. concluded that IVT+Laser is better than IVB alone in reducing CMT, which went against the findings of Lam et al. who indicated that IVT+Laser has no better CMT reduction at 24 weeks than IVT alone; and laser alone was significantly worse than the 2 aforementioned groups [55, 56]. Surprisingly, Preti et al. concluded that IVB+Laser may significantly increase the thickness compared with laser alone at a 4-week follow-up, however, they investigated macular choroidal thickness and not CMT [57]. It was proposed that IVT+laser might be superior due to its coupled anti-inflammatory and anti-angiogenic effects [13]. However, this could only affect the CMT reduction, but it does not guarantee an improvement in VA. Also, no cumulative analysis of these trials can yield statistical significance. They suffer from a critical degree of bias and heterogeneity: no description of allocation concealment, blinding of participants, or blinding of outcome assessment were reported in the work of Pappas et al. Comparatively, a high risk of blinding of participants was found in the report of Lam et al; a relatively small sample size, very short follow-up and unjustified inclusion of patients with and without DME were detected in the work of Preti et al. Moreover, clinicians should reconsider the IVB and IVT combination with laser for a further investigation.

The quality of a systematic review and meta-analysis rests upon the qualities of its included studies. Our included studies exhibit relatively high quality. Our findings settle a group of assumptions and provide a reliable reference for future clinical decisions. To our knowledge, this is the first systematic review and meta-analysis that analyzes the long-term outcomes of IVB and IVT over 48 weeks and provide a meta-regression for injections, pathogenesis, and interaction between VA & CMT. Even so, there were some limitations to our work. The results in CMT at 6, 12, 24, 48 weeks were limited by the heterogeneity of the included studies. Likewise, only two studies investigated IVT vs. IVB/IVT, making it difficult to conclude anything definitive on this aspect. The variations in the clinical definitions and subtypes of DME may contribute to the clinical heterogeneity. Additionally, most of the included trials had relatively small sample sizes.

Our analysis reveals that repeated IVB injections associate with better VA. Overall, the current evidence indicates that IVB is superior to IVT and IVB+IVT in improving VA for DME patients up to 48 weeks. The combined IVB+IVT does not seems to be more promising or beneficial. CMT reduction appears to be comparable across the three arms (IVB vs. IVT, IVB vs. IVB+IVT, and IVT vs. IVB+IVT) in short and long-terms. It is in favor of IVB when it comes to the significant reduction of IOP and the avoidance of IOH compared to IVT and IVB+IVT. Further multi-center, large sample RCTs are needed to investigate the efficacy of laser photocoagulation combined with IVB versus laser combined with IVT. Future studies should consider accurate reporting of pathogenic markers across groups and subgroups to allow for a better understanding of DME's underlying pathogenesis.

## Supporting information

S1 Checklist PRISMA 2009 checklist. (DOC)

S1 Fig Risk of bias summary; (+) indicates low risk of bias, (?) indicates unclear risk of bias, and (-) indicates high risk of bias. (TIF)

S2 Fig A. The overall meta-regression mean difference of the interaction between each pathogenic factor on x-axis and CMT on y-axis. The diamond indicates significant prediction. B. The overall meta-regression mean difference of the interaction between each pathogenic factor on x-axis and BCVA on y-axis. The diamond indicates significant prediction. C. The overall meta-regression mean difference of the interaction between each pathogenic factor on x-axis and IOP on y-axis. The diamond indicates significant prediction. D. The overall meta-regression mean difference of the interaction between number of injections on x-axis and each outcome on y-axis. The diamond indicates significant prediction.
(TIF)

S1 File Quality assessment of RCTs. (DOCX)

S2 File (DOCX)
