# Supplementary figures and images for: Intravitreal bevacizumab versus intravitreal triamcinolone for diabetic macular edema–Systematic review, meta-analysis and meta-regression
Source: PLoS One. 2021 Jan 12;16(1):e0245010. doi: 10.1371/journal.pone.0245010 (PMC7802957; doi:10.1371/journal.pone.0245010)

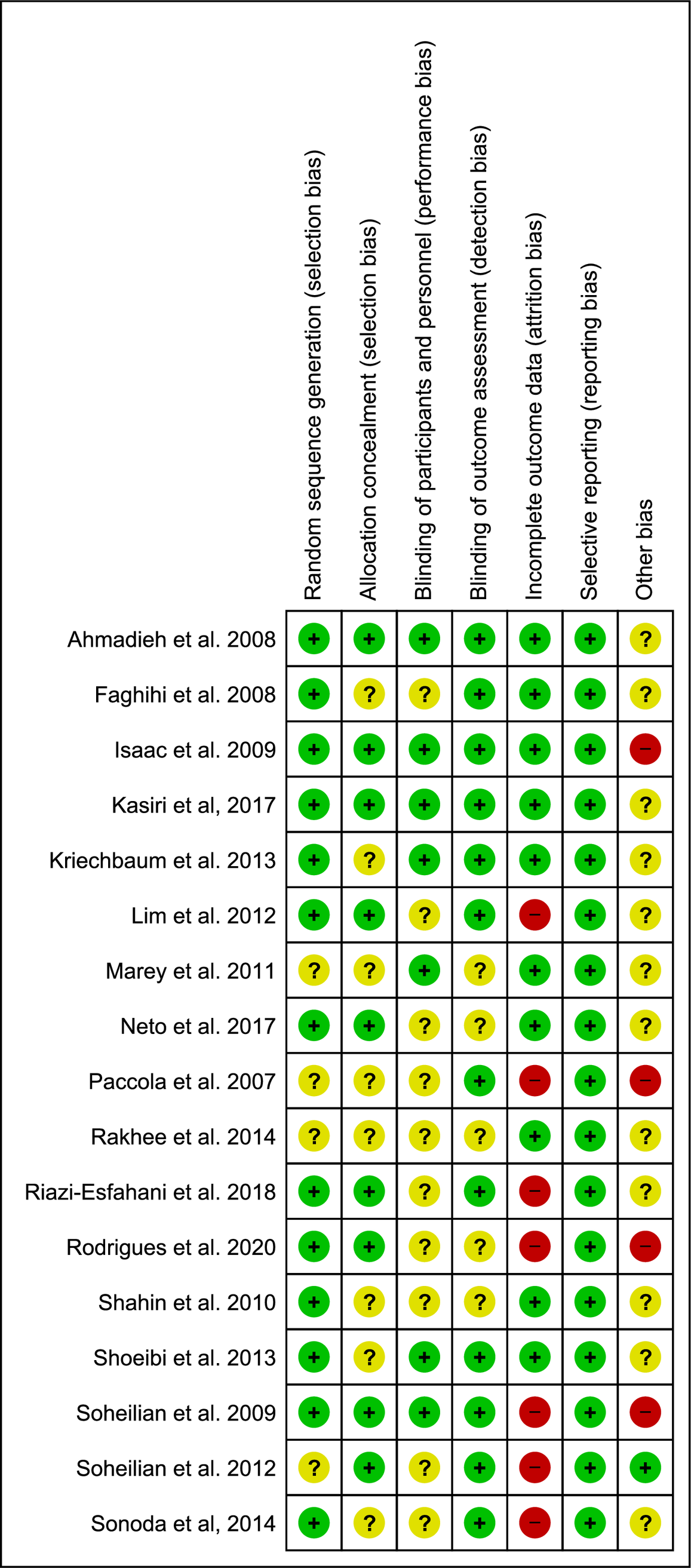

Supplement: S1 Fig — (TIF) [file pone.0245010.s002.tif]

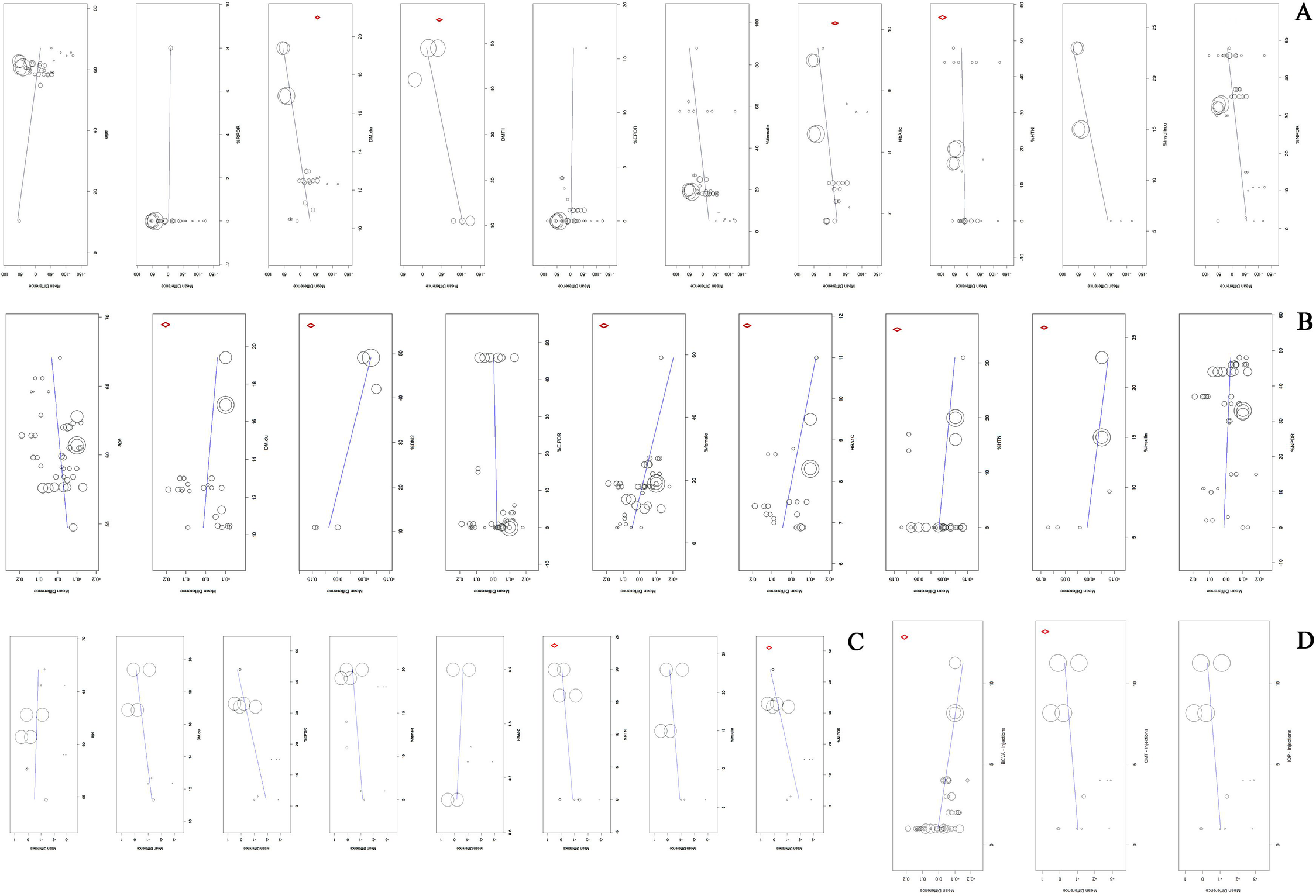

Supplement: S2 Fig — A. The overall meta-regression mean difference of the interaction between each pathogenic factor on x-axis and CMT on y-axis. The diamond indicates significant prediction. B. The overall meta-regression mean difference of the interaction between each pathogenic factor on x-axis and BCVA on y-axis. The diamond indicates significant prediction. C. The overall meta-regression mean difference of the interaction between each pathogenic factor on x-axis and IOP on y-axis. The diamond indicates significant prediction. D. The overall meta-regression mean difference of the interaction between number of injections on x-axis and each outcome on y-axis. The diamond indicates significant prediction. (TIF) [file pone.0245010.s003.tif]
