# Supplementary material for: Intravitreal bevacizumab versus intravitreal triamcinolone for diabetic macular edema–Systematic review, meta-analysis and meta-regression
Source: PLoS One. 2021 Jan 12;16(1):e0245010. doi: 10.1371/journal.pone.0245010 (PMC7802957; doi:10.1371/journal.pone.0245010)
Supplement: S1 File — (DOCX) [file pone.0245010.s004.docx]

S1 File. Quality assessment of RCTs.

| **Author judgment** | **Risk of bias** | **Rodrigues et al. 2020** |  |
| --- | --- | --- | --- |
| The randomization was determined from a binomial distribution by JMP statistical software. | Low risk | Random sequence generation (selection bias) |  |
| The randomization was determined from a binomial distribution by JMP statistical software, and then the main investigator followed a sequence to allocate the included subjects. | Low risk | Allocation concealment (selection bias) |  |
| Not reported. | Unclear | Blinding of participants and personnel (performance bias) |  |
| Not reported. | Unclear | Blinding of outcome assessment (detection bias) |  |
| Week 24: (9 patients/11 eyes) were lost follow-up.  Week 28: One eye from each group (2 patients/3 eyes) was lost to follow-up.  Week 32: One patient (1 eye) withdrew from the study after the.  Week36: 2patients/2eyesfromgroupIIand2patients/2eyes from group III were lost to follow-up. | High risk | Incomplete outcome data (attrition bias) |  |
| Outcomes listed in the methods section are reported in the result section. | Low risk | Selective reporting (reporting bias) |  |
| The project had financial support by CNPq government (National Research Council) grant number 142177/2016-4 | High risk | Other bias |  |
| **Author judgment** | **Risk of bias** | **Riazi-Esfahaniet al. 2018** |  |
| Randomization was performed by a random block permutation method according to a computer-generated randomization list. | Low risk | Random sequence generation (selection bias) |  |
| Random allocation sequencing was performed by a biostatistician. Details of the series were unknownbythe study investigators. | Low risk | Allocation concealment (selection bias) |  |
| Not reported. | Unclear | Blinding of participants and personnel (performance bias) |  |
| At each visit, visual acuitywas determined, and OCTwas done by certiﬁed examiners masked to both the randomization and the ﬁndings of previous measurements. | Low risk | Blinding of outcome assessment (detection bias) |  |
| Main limitations of this study were number of cases and relatively high loss to follow-up due to different causes. | High risk | Incomplete outcome data (attrition bias) |  |
| Outcomes listed in the methods section are reported in the result section. | Low risk | Selective reporting (reporting bias) |  |
|  | Unclear | Other bias |  |
| **Author judgment** | **Risk of bias** | **Netoet al. 2017** |  |
| This study was a multicenter, masked, and randomized clinical trial. The study participants were randomly assigned into groups of 30 subjects followed by a process of simple randomization with cards with equal probability (1:1:1) and distributed to one of the three treatment groups. | Low risk | Random sequence generation (selection bias) |  |
| The study participants were randomly assigned into groups of 30 subjects followed by a process of simple randomization with cards with equal probability (1:1:1) and distributed to one of the three treatment groups. | Low risk | Allocation concealment (selection bias) |  |
| Not reported. | unclear | Blinding of participants and personnel (performance bias) |  |
| Not reported. | unclear | Blinding of outcome assessment (detection bias) |  |
| There is no incomplete outcome data. | Low risk | Incomplete outcome data (attrition bias) |  |
| Outcomes listed in the methods section are reported in the result section. | Low risk | Selective reporting (reporting bias) |  |
| This study was supported in part by Sao Paulo Research Foundation. | Unclear | Other bias |  |
| **Author judgment** | **Risk of bias** | **Kasiri et al, 2017** |  |
| Randomization was performed using the randomly permuted blocks according to a computer generated randomization list. | Low risk | Random sequence generation (selection bias) | |
| Details of the series were unknown to the investigators. | Low risk | Allocation concealment (selection bias) | |
| For complete masking, subjects were prevented from seeing the syringe. | Low risk | Blinding of participants and personnel (performance bias) | |
| The outcome assessors (optometrists responsible for visual acuity and OCT testing) and data analysts were masked to the allocation. | Low risk | Blinding of outcome assessment (detection bias) | |
| Data is recorded for all patients. | Low risk | Incomplete outcome data (attrition bias) | |
| Outcomes listed in the methods section are reported in the result section. | Low risk | Selective reporting (reporting bias) | |
|  | Unclear | Other bias | |
| **Author judgment** | **Risk of bias** | **Sonoda et al, 2014** | |
| Randomization was done by sealed envelopes after the patient was found to be eligible. | Low risk | Random sequence generation (selection bias) | |
| Not reported. | Unclear | Allocation concealment (selection bias) | |
| Not reported. | Unclear | Blinding of participants and personnel (performance bias) | |
| Measurement of datawas done in a masked fashion by two examiners. The remaining investigators were not permitted access to any information on the outcome before completion of the analyses. | Low risk | Blinding of outcome assessment (detection bias) | |
| Four patient were lost to follow-up and one dropped out 2because of treatment failure. | High risk | Incomplete outcome data (attrition bias) | |
| Outcomes listed in the methods section are reported in the result section. | Low risk | Selective reporting (reporting bias) | |
|  | Unclear | Other bias | |
| **Author judgment** | **Risk of bias** | **Rakheeet al. 2014** | |
| It is a prospective, randomized, comparative interventional case series of 98 eyes of 81 subjects with diabetic macular edema but randomization method was not reported. | Unclear | Random sequence generation (selection bias) | |
| Not reported. | Unclear | Allocation concealment (selection bias) | |
| Not reported. | Unclear | Blinding of participants and personnel (performance bias) | |
| Not reported. | Unclear | Blinding of outcome assessment (detection bias) | |
| No missing data points. | Low risk | Incomplete outcome data (attrition bias) | |
| All pre specified outcomes were reported. | Low risk | Selective reporting (reporting bias) | |
|  | Unclear | Other bias | |
| **Author judgment** | **Risk of bias** | **Shoeibi et al. 2013** | |
| Randomization was performed using the randomly permuted blocks according to a computer generated randomization list. | Low risk | Random sequence generation (selection bias) | |
| A random allocation sequence was performed by a biostatistician. Details of the series were unknown to the investigators. | Low risk | Allocation concealment (selection bias) | |
| Not reported. | Unclear | Blinding of participants and personnel (performance bias) | |
| The outcome assessors (optometrists responsible for visual acuity and OCT testing) and data analysts were masked to the allocation. | Low risk | Blinding of outcome assessment (detection bias) | |
| No missing data points. | Low risk | Incomplete outcome data (attrition bias) | |
| Outcomes listed in the methods section are reported in the result section. | Low risk | Selective reporting (reporting bias) | |
|  | Unclear | Other bias | |
| **Author judgment** | **Risk of bias** | **Kriechbaum et al. 2013** | |
| The study was designed as prospective, randomized, double-masked, comparative interventional case series. | Low risk | Random sequence generation (selection bias) | |
| Not reported. | Unclear | Allocation concealment (selection bias) | |
| Double-masked study. | Low risk | Blinding of participants and personnel (performance bias) | |
| Double-masked study. | Low risk | Blinding of outcome assessment (detection bias) | |
| No missing data points. | Low risk | Incomplete outcome data (attrition bias) | |
| Outcomes listed in the methods section are reported in the result section. | Low risk | Selective reporting (reporting bias) | |
|  | Unclear | Other bias | |
| **Author judgment** | **Risk of bias** | **Lim et al. 2012** | |
| Randomization was performed using a random block permutation method according to a computer-generated randomization list. | Low risk | Random sequence generation (selection bias) | |
| The random allocation sequence was performed by a biostatistician. Details of the series were unknown to the investigators. | Low risk | Allocation concealment (selection bias) | |
| Not reported. | Unclear | Blinding of participants and personnel (performance bias) | |
| Visual acuity assessment and OCT were performed by an optometrist who was blinded to the group status of the patients. In addition, the statistician who performed the analysis was also blinded to the details of the series. | Low risk | Blinding of outcome assessment (detection bias) | |
| 6 eyes had incomplete data because of loss to follow-up and 3 eyes refused to continue intervention. | High risk | Incomplete outcome data (attrition bias) | |
| Outcomes listed in the methods section are reported in the result section. | Low risk | Selective reporting (reporting bias) | |
|  | Unclear | Other bias | |
| **Author judgment** | **Risk of bias** | **Soheilian et al. 2012** | |
| Randomized trial but randomization method was not reported. | low risk | Random sequence generation (selection bias) | |
| Eligible eyes had been randomly allocated to 1 of 3 study groups. | low risk | Allocation concealment (selection bias) | |
| Not reported. | low risk | Blinding of participants and personnel (performance bias) | |
| All procedures were run by staff members other than the study investigators to preserve investigator masking. | low risk | Blinding of outcome assessment (detection bias) | |
| 32 patients (37 eyes) did not complete their follow-ups up to 24 months. | High risk | Incomplete outcome data (attrition bias) | |
| Outcomes listed in the methods section are reported in the result section. | Low risk | Selective reporting (reporting bias) | |
| Supported in part by Ophthalmic Research Center of Shahid Beheshti, University of Medical Sciences, Tehran, Iran. | High risk | Other bias | |
| **Author judgment** | **Risk of bias** | **Marey et al. 2011** | |
| Patients were divided randomly into three study groups but randomization method was not reported. | Unclear | Random sequence generation (selection bias) | |
| Not reported. | Unclear | Allocation concealment (selection bias) | |
| For complete masking, a needleless syringe was pressed against the globe at the super temporal quadrant in groups I and III; and all injections were done once and not repeated in the follow-up period. | Low risk | Blinding of participants and personnel (performance bias) | |
|  | Unclear | Blinding of outcome assessment (detection bias) | |
| No missing data points. | Low risk | Incomplete outcome data (attrition bias) | |
| All prespecified outcomes were reported. | Low risk | Selective reporting (reporting bias) | |
|  | Unclear | Other bias | |
| **Author judgment** | **Risk of bias** | **Shahin et al. 2010** | |
| The study cohort was divided into two groups using a randomization schedule. | Low risk | Random sequence generation (selection bias) | |
| Not reported | Unclear | Allocation concealment (selection bias) | |
| Not reported | Unclear | Blinding of participants and personnel (performance bias) | |
| Not reported | Unclear | Blinding of outcome assessment (detection bias) | |
| No missing data points. | Low risk | Incomplete outcome data (attrition bias) | |
| All prespecified outcomes were reported. | Low risk | Selective reporting (reporting bias) | |
|  | Unclear | Other bias | |
| **Author judgment** | **Risk of bias** | **Isaac et al. 2009** | |
| This is a prospective, double-blind, interventionist, comparative and randomized study. The drawing to randomize the patients to the drug which would be injected in one of the eyes was conducted by someone not participating in the study. | Low risk | Random sequence generation (selection bias) | |
| The drawing to randomize the patients to the drug which would be injected in one of the eyes was conducted by someone not participating in the study. | Low risk | Allocation concealment (selection bias) | |
| Double-blind study. | Low risk | Blinding of participants and personnel (performance bias) | |
| Double-blind study. | Low risk | Blinding of outcome assessment (detection bias) | |
| No missing data points. | Low risk | Incomplete outcome data (attrition bias) | |
| All prespecified outcomes were reported. | Low risk | Selective reporting (reporting bias) | |
| There was no separation between patients who had already received previous macular photocoagulation from treatment naive patients. | High risk | Other bias | |
| **Author judgment** | **Risk of bias** | **Soheilian et al. 2009** | |
| Randomization was performed using the random block permutation method according to a computer-generated randomization list. | Low risk | Random sequence generation (selection bias) | |
| Random allocationsequence was performed by a biostatistician. The detail of serieswas unknown by the study investigators. | low risk | Allocation concealment (selection bias) | |
| Injection was done by aneedleless syringe pressed against the conjunctiva. To keep themasking process, patients were prevented from seeing the syringes. | Low risk | Blinding of participants and personnel (performance bias) | |
| All procedures were run by staff members other than the study investigators to preserve investigator masking. Best-corrected  VA measurement and OCT were performed by certified examiners masked both to the randomization and to the findings of previous measurements. | Low risk | Blinding of outcome assessment (detection bias) | |
| Missing dataespecially in thecombined treatment group. | High risk | Incomplete outcome data (attrition bias) | |
| All pre-specified outcomes were reported. | Low risk | Selective reporting (reporting bias) | |
| Supported by the Ophthalmic Research Center of Shahid Beheshti University (MC) Tehran, Iran. | High risk | Other bias | |
| **Author judgment** | **Risk of bias** | **Ahmadieh et al. 2008** | |
| Randomization was performed using a random block permutation method according to a computer-generated randomization list. | Low risk | Random sequence generation (selection bias) | |
| A random allocation sequence was performed by a biostatistician. Details of the series were unknown to the investigators. | Low risk | Allocation concealment (selection bias) | |
| Subjects were masked to the treatment modality. | Low risk | Blinding of participants and personnel (performance bias) | |
| Visual acuity assessment and OCT were performed by optometrists who were masked to the groups. | Low risk | Blinding of outcome assessment (detection bias) | |
| No missing data. | Low risk | Incomplete outcome data (attrition bias) | |
| All pre-specified outcomes were reported. | Low risk | Selective reporting (reporting bias) | |
|  | Unclear | Other bias | |
| **Author judgment** | **Risk of bias** | **Faghihi et al. 2008** | |
| Eyes were randomly assigned via balanced blocked randomization. | Low risk | Random sequence generation (selection bias) | |
| Not reported. | Unclear | Allocation concealment (selection bias) | |
| Not reported. | Unclear | Blinding of participants and personnel (performance bias) | |
| An ophthalmologist who was masked about treatment arms performed final assessment. | Low risk | Blinding of outcome assessment (detection bias) | |
| No missing data. | Low risk | Incomplete outcome data (attrition bias) | |
| All pre-specified outcomes were reported. | Low risk | Selective reporting (reporting bias) | |
|  | Unclear | Other bias | |
| **Author judgment** | **Risk of bias** | **Paccola et al. 2007** | |
| Randomized prospective study but randomization method was not reported | Unclear | Random sequence generation (selection bias) | |
| Not reported. | Unclear | Allocation concealment (selection bias) | |
| Not reported. | Unclear | Blinding of participants and personnel (performance bias) | |
| Study data were collected, interpreted and analyzed by two other masked investigators. | Low risk | Blinding of outcome assessment (detection bias) | |
| Twenty-six of the 28 patients were ultimately included in the analyses (two patients missed two consecutive study visits and were excluded from analyses). | High risk | Incomplete outcome data (attrition bias) | |
| All pre-specified outcomes were reported. | Low risk | Selective reporting (reporting bias) | |
| Supported in part by Conselho Nacional de Desenvolvimento Cientı ́fico eTecnolo ́gico (CNPq), grant no. 302940/2005-7 | High risk | Other bias | |
