# Supplementary material for: Intravitreal bevacizumab versus intravitreal triamcinolone for diabetic macular edema–Systematic review, meta-analysis and meta-regression
Source: PLoS One. 2021 Jan 12;16(1):e0245010. doi: 10.1371/journal.pone.0245010 (PMC7802957; doi:10.1371/journal.pone.0245010)
Supplement: S2 File — (DOCX) [file pone.0245010.s005.docx]

Full electronic Boolean search:

(("diabetic macular edema" OR "DME") AND ("bevacizumab" OR "Avastin" OR "triamcinolone" OR "IVB" OR "IVT"))
